# Comparison of the mRNA expression profile of B‐cell receptor components in normal CD5‐high B‐lymphocytes and chronic lymphocytic leukemia: a key role of ZAP70

**DOI:** 10.1002/cam4.1257

**Published:** 2017-11-10

**Authors:** Aleena A. Gladkikh, Daria M. Potashnikova, Victor Tatarskiy, Margarita Yastrebova, Alvina Khamidullina, Natasha Barteneva, Ivan Vorobjev

**Affiliations:** ^1^ Biological Department M.V. Lomonosov Moscow State University Moscow Russia; ^2^ N.N. Blokhin Russian Cancer Research Center Moscow Russia; ^3^ Department of Pediatrics Harvard Medical School Boston Massachusetts; ^4^ Department of Biology School of Science and Technology Nazarbayev University Astana Kazakhstan; ^5^ A.N. Belozersky Institute of Physico‐Chemical Biology M.V. Lomonosov Moscow State University Moscow Russia

**Keywords:** Cancer biology, lymphoma, B‐cell receptor, Zap‐70

## Abstract

The B‐cell receptor (BCR) signaling pathway is of great importance for B‐cell survival and proliferation. The BCR expressed on malignant B‐CLL cells contributes to the disease pathogenesis, and its signaling pathway is currently the target of several therapeutic strategies. Although various BCR alterations have been described in B‐CLL at the protein level, the mRNA expression levels of tyrosine kinases in B‐CLL compared to that in normal CD5‐high and CD5‐low B‐lymphocytes remain unknown. In the current study, we measured the mRNA expression levels of *CD79A*,* CD79B*,* LYN*,* SYK*,* SHP1*, and *ZAP70* in purified populations of CD5‐high B‐CLL cells, CD5‐low B‐cells from the peripheral blood of healthy donors, and CD5‐high B‐cells from human tonsils. Here, we report a clear separation in the B‐CLL dataset between the *ZAP70*‐high and *ZAP70*‐low subgroups. Each subgroup has a unique expression profile of BCR signaling components that might reflect the functional status of the BCR signaling pathway. Moreover, the *ZAP70‐*low subgroup does not resemble either CD5‐high B‐lymphocytes from the tonsils or CD5‐low lymphocytes from PBMC (*P* < 0.05). We also show that *ZAP70* is the only gene that is differentially expressed in CD5‐high and CD5‐low normal B‐lymphocytes, confirming the key role of Zap‐70 tyrosine kinase in BCR signaling alterations in B‐CLL.

## Introduction

B‐cell chronic lymphocytic leukemia (B‐CLL) is the most common type of leukemia in Western countries, and the majority of patients are >60 years of age at diagnosis 1. Over the last decades, the understanding of B‐CLL biology has advanced considerably with the discovery of alterations in the B‐cell signaling transduction pathways that contribute to the disease heterogeneity and help predict its clinical course.

All malignant B‐CLL cells share several unique features, including the absence of a common molecular abnormality, randomly balanced chromosomal translocations, and the inability of malignant B‐lymphocytes to exist as a transformed cell line 2, 3. Most intriguingly, however, nearly all B‐CLL cells express CD5 4, which is a common T‐cell surface antigen, and it remains unclear whether this feature is a fingerprint of the normal cellular counterpart or a malignancy‐related property. After the discovery of the CD5‐high B‐CLL phenotype, Hayakawa et al. 5 demonstrated the presence of CD5‐high B‐cells in fetal livers and their preferential abundance in young mice. Similarly, CD5‐high B‐cells are more frequently observed in human cord blood than in adult peripheral blood 6, and their percentage decreases with age. In adult human lymphoid tissue, CD5‐high B‐cells are located in the follicle mantle zone and comprise a large subpopulation of tonsillar B‐cells. In a mouse model, it has recently been shown that a subset of CD5‐high cells has a potential to give rise to B‐CLL‐like cells in adult animals 7.

Despite the shared pattern of surface antigens, subpopulations of B‐CLL cells demonstrate a striking heterogeneity in their molecular and functional features. Thus, B‐CLL cells in the peripheral blood have a genetic profile and phenotype that are similar to those of memory B‐cells, while B‐CLL cells in the lymph node and bone marrow exhibit the characteristics of activated B‐cells with an increased proliferative potential 8, 9, 10. At the molecular level, the diversity of B‐CLL clones, which are known to contribute to disease pathogenesis, affect the BCR signaling pathway.

The B‐cell receptor is a transmembrane immunoglobulin complex that is associated with an Ig*α* (CD79a) and Ig*β* (CD79b) heterodimer. In normal B‐cells, tyrosine kinases, such as Lyn and Syk, phosphorylate the ITAM motifs in the CD79*α* and CD79*β* receptor subunits, resulting in the downstream activation of BTK, PI3K, and PLC*γ* and further signal propagation 11. BCR abnormalities in B‐CLL cells include low to undetectable levels of monoclonal surface immunoglobulins, a reduced expression of CD79b, and a malfunction in the downstream pathway, which is predicated by the constitutive activation of both the Lyn and Syk kinases 12, 13. The constitutive activation of Lyn leads to the phosphorylation of the immunoreceptor tyrosine inhibitory motifs (ITIMs) in CD5 inhibitory coreceptors, which are aberrantly expressed on B‐CLL cells. Thus, CD5 provides an anchoring site for Src homology 2 domain‐containing phosphatase 1 (Shp‐1), thereby triggering negative feedback signaling. In addition, compared to normal B‐cells, Syk tyrosine kinase has been reported to be overexpressed in B‐CLL cells at both the mRNA and protein levels 14. However, the most obscure feature of B‐CLL signaling is the expression of Zap‐70 tyrosine kinase in malignant lymphocytes. Zap‐70 is normally present in T‐cells and B‐CLL cells and is thought to reflect the BCR activation status, which, in turn, correlates with increased tumor proliferation and a shorter time to disease progression 13, 15. Altogether, these findings implicate the antigen‐dependent BCR activation as a major pathway of B‐CLL progression and pathogenesis 16.

Although it is known that Zap‐70 can be expressed in a subpopulation of normal CD5‐high tonsillar B‐cells depending on the state of their activation, the BCR status and signaling transduction pathways in these unconventional B‐lymphocytes remain to be elucidated. In this work, we describe for the first time the transcriptional profiles of BCR signaling components in CD5‐high and CD5‐low normal B‐cells, compare normal B‐cells to malignant B‐CLL lymphocytes, and confirm the role of *ZAP70* as a unique kinase gene that allows for the distinction among different normal and tumor B‐cell subpopulations.

## Materials and Methods

### Samples

The B‐CLL specimens were obtained from untreated patients undergoing lymphoma diagnosis verification at the National Research Centre for Haematology (Moscow) or “GeneTechnology” Diagnostic Centre (Moscow). The samples were immunophenotyped by flow cytometry for each patient. Peripheral blood from healthy donors (*n* = 10) was used for the normal T‐ and B‐lymphocyte sorting. Additionally, 10 specimens of nonmalignant tonsils from children undergoing routine tonsillectomies were obtained from St. Vladimir's Children Hospital (Moscow, Russia). All tissue specimens were evaluated by immunohistochemistry; and single cell suspensions were prepared using a MediMachine (BD, USA) for the flow cytometry and CD5+CD19+ B‐cell sorting. All human material was obtained with informed consent. All procedures were in accordance with the ethical standards of the responsible local committee (at the National Research Centre for Haematology) and the Helsinki Declaration of 1975 as revised in 2008.

### Flow cytometry

PBMCs of B‐CLL patients and healthy donors were derived from whole blood by Ficoll gradient centrifugation. Single cell suspensions were prepared from fresh human tonsil specimens using a MediMachine (BD, USA) and filtered through a 40‐*μ*m cell filter. Surface staining was performed with the monoclonal antibodies anti‐CD5 FITC (clone DK23, DAKO, USA), anti‐CD19 PE‐Cy5 (clone HIB19, eBioscience, USA), anti‐CD23 PE (clone MHM6, DAKO, USA), FMC7 FITC (clone MCA792F, Serotec, USA), anti‐CD20 PE‐Cy5 (clone 2H7, eBioscience, USA), and anti‐*κ* light Ig chain and anti‐*λ* light Ig chain (*κ*‐FITC/*λ*‐PE, AHM4907, Invitrogen, USA). The cells were incubated with the antibodies at +4°C for 20 min, washed with PBS, and analyzed on a FACSAria SORP cell sorter (BD Biosciences, USA). Diva software (BD Biosciences, USA) was employed for the data analysis.

All samples were kept at +4°C during staining and prior to FACS analysis. Primary FACS profiles were obtained for each specimen on the day of retrieval typically in 4 h after extraction. Aliquots of cells were frozen in FBS with 10% DMSO in liquid nitrogen for further FACS sorting.

### Cell sorting

Sorting was performed separately on thawed specimens using a 5‐laser FACSAria SORP instrument (BD Biosciences, USA) with a 70‐*μ*m nozzle and the corresponding system pressure parameters. Over 10,000 cells were sorted in each sample. The purity of the sorted populations was assessed by flow cytometry and exceeded 98%.

B‐CLL cells were stained with anti‐CD3‐APC‐Cy7 (clone HIT3a, BioLegend, USA), anti‐CD23‐APC (EBVCS‐5, BioLegend, USA), anti‐CD19‐FITC (clone HIB19, eBioscience, USA), anti‐CD5‐PE‐Cy7 (clone UCHT2, BD, USA), and anti‐CD38‐PE or anti‐CD38‐PE‐Cy5 (both HIT2, eBioscience, USA) antibody combination. Malignant B‐CLL cells were purified from peripheral blood based on the CD5+/CD3−/CD19+/CD23+/CD38+ ‐ immunophenotype.

CD5‐high normal tonsillar B‐lymphocytes were stained with anti‐CD3‐FITC (clone HIT3a, BioLegend, USA), anti‐CD23‐PE (clone EBVCS‐5, BioLegend, USA), anti‐CD19‐PerCP‐Cy5.5 (clone HIB19, eBioscience, USA), anti‐CD5‐APC (clone UCHT2, BD, USA), and anti‐CD38‐PE‐Cy7 (clone HIT2, eBioscience, USA) antibody combination. Normal CD5‐high B‐cells with the CD5+/CD3−/CD19+/CD23−/CD38+ phenotype were purified from human tonsil suspensions. To set gate on tonsillar CD5‐high B‐cells, FMO control for CD5 (anti‐CD3‐FITC (clone HIT3a, BioLegend, USA), anti‐CD23‐PE (clone EBVCS‐5, BioLegend, USA), anti‐CD19‐PerCP‐Cy5.5 (clone HIB19, eBioscience, USA), and anti‐CD38‐PE‐Cy7 (clone HIT2, eBioscience, USA) antibody combination) was used.

Peripheral blood B‐ and T‐cells from healthy donors were stained with anti‐CD3‐FITC (clone HIT3a, BioLegend, USA), anti‐CD19‐PerCP‐Cy5.5 (clone HIB19, eBioscience, USA), and anti‐CD45‐PE (clone HI30, BioLegend, USA) antibody combination. B‐ and T‐cells were sorted according to the CD3−/CD19+/CD45+ and CD3+/CD19−/CD45+ phenotypes, respectively. Sorted cells were sedimented at 1000*g* and lysed for RNA extraction immediately.

### Zap‐70 flow cytometry

Three antibody clones against Zap‐70 (SBZAP, 2F3.2, and 1E7.2) were tested for flow cytometry and Western blot. Anti‐Zap‐70‐PE (SBZAP) was further used for flow cytometry. Cells were fixed with 1% paraformaldehyde (Merck, Germany) for 5 min, washed once with PBS, and permeabilized with 1X Perm II reagent (BD, USA) according to the manufacturer's instructions. The staining panel included either anti‐CD3‐FITC (clone HIT3a, BioLegend), anti‐Zap‐70‐PE (clone SBZAP, Beckman Coulter) and anti‐CD22‐APC (clone S‐HCL‐1, BD), or anti‐CD3‐PE‐Cy7 (clone UCHT1, BioLegend), anti‐CD19‐FITC (clone HIB19, eBioscience), anti‐Zap‐70‐PE (clone SBZAP, Beckman Coulter), and anti‐CD22‐APC (clone S‐HCL‐1, BD).

### RNA and cDNA

RNA was extracted from thawed suspensions of the sorted and unsorted cells using the RNeasy Mini Kit (Qiagen, USA) according to the manufacturer's instructions. The RNA concentration was measured using a NanoPhotometer (Implen, Germany), and its purity was assessed according to the A260/A280 and A260/A230 ratios. cDNA was transcribed using the ImProm‐II AMV‐Reverse Transcription Kit (Promega, USA) according to the manufacturer's instructions.

### Primers and real‐time PCR

Real‐time qPCR was further performed on Quantica (Barlow Scientific, UK) and StepOne (Applied Biosystems, USA) cyclers using Taq‐polymerase in SYBR Green I buffer (Syntol, Russia). The reaction protocol included denaturation (95°C, 10 min), followed by 40 amplification cycles (95°C, 15 sec; 60°C, 30 sec; and 72°C, 60 sec). All samples were processed in triplicate. All primers were synthesized and HPLC‐purified by Syntol (Russia). A control cDNA sample was included in each PCR run and served as an inter‐run calibrator (IRC) to standardize the data. The primer sequences are listed in Table S2.

### Data normalization

qPCR data were normalized according to the method proposed by Vandesompele et al. 17. The following three reference genes were used for the normalization: *YWHAZ*,* UBC*, and *HPRT1*. In short, the relative expression value of each gene of interest was divided by the geometric mean of the reference genes’ relative expression values. Three suitable reference genes were established previously 18 and used in the current work.

### Western blotting

Cells were lyzed in RIPA buffer (50 mmol/L Tris‐HCl pH 7.4, 1% NP‐40, 0.1% SDS, 150 mmol/L NaCl, 1 mmol/L EDTA, 0.5% sodium deoxycholate, protein inhibitor cocktail [Roche], and 2 mmol/L PMSF) for 30 min on ice. Protein concentration was measured using Bradford reagent (Sigma, USA). Thirty *μ*g of protein lysates were resolved in 12% SDS‐PAGE gel and transferred to 0.2 *μ*m nitrocellulose membrane (GE Healthcare, USA), blocked with 5% nonfat dry milk, 1X TBS, 0.1% Tween‐20 for 40 min at RT. Membranes were incubated with primary antibodies against Syk (clone Syk‐01, Abcam, UK), Lyn (clone Lyn‐01, Abcam, UK), SHP1 (clone EPR5519, Abcam, UK), Zap70 (clone 2F3.2, Invitrogen, USA), and Histone‐3 (Cell Signaling) at +4°C overnight in 1% BSA in 1X TBS, 0.1% Tween‐20. After washing membranes were incubated with secondary antibodies linked with mouse or rabbit horseradish peroxidase (Cell Signaling, USA) for 1 h at RT. Membranes were washed again, and the proteins were visualized using Image Quant LAS 4000 system (GE Healthcare, USA).

### Statistical analysis

The statistical analysis and data plotting were performed using GraphPad Prism (GraphPad Software, version 5, San Diego, CA, USA). The differences in the mRNA expression levels between groups of malignant and normal lymphocytes were considered significant based on a nonparametric Mann–Whitney test with Bonferroni correction for multiple testing.

## Results

### Purification of normal lymphocytes from tonsils

**Figure 1:**
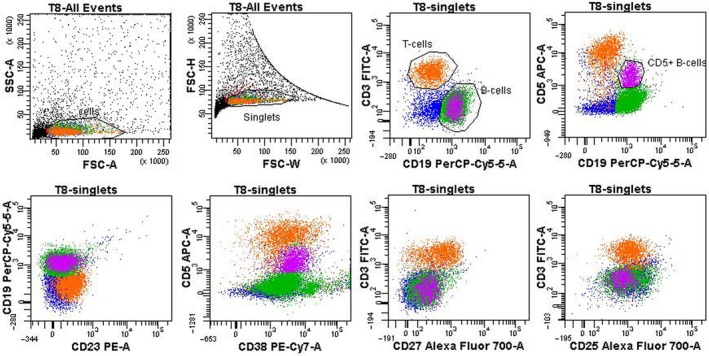
Flow cytometry and sorting strategy for CD5+ B‐cells from human tonsils. The upper panel presents the sorting gates. “Cells” are gated from debris according to forward and side light scatter characteristics; “singlets” are gated according to FSC signal width/height ratio; “B‐cells” are gated based on CD19 fluorescence; a subpopulation of B‐cells (“CD5+ B‐cells”) with a moderate CD5+ staining level; the gating is sequential. To gate on the subpopulation of “CD5+ B‐cells” correctly FMO control was used (see Materials and Methods). CD5 median fluorescence values (MFI) for different lymphocyte populations are as follows: MFI(CD5) T‐cells = 9462, MFI(CD5) B‐cells(general) = 405, MFI(CD5)CD5+B‐cells = 1939. The lower panel expands on CD5+ B‐cell phenotype: unlike B‐CLL the cells of interest are CD23‐low, CD27‐low, CD25‐low, and universally CD38+.

Nonmalignant CD5‐high B‐cells with the CD5+/CD19+/CD23‐low−/CD38+ phenotype were purified by FACS from human tonsils (*n* = 10) and comprised 3–10% B‐lymphocytes. The normal B‐ (*n* = 10) and T‐cell (*n* = 10) samples were sorted from healthy donor peripheral blood based on the CD19+/CD45+ and CD3+/CD45+ phenotypes, respectively. The sorting strategy for the minor normal population of CD5+/CD19+ tonsillar B‐cells is presented in Figure 1.

All sorted populations were reanalyzed by flow cytometry to confirm a purity >98%, and 10^4^–10^5^ cells were collected from each sample. All samples had a similar CD5‐low/CD19+ phenotype. Unlike the B‐CLL lymphocytes, these cells did not express CD23, CD43, CD25, or CD27 on their surface.

### Cell sorting of B‐CLL samples is a prerequisite for a gene expression analysis

The differential diagnosis of B‐CLL was established according to the WHO classification as follows: the clonality of the neoplastic lymphocytes was confirmed by both surface and intracellular kappa/lambda immunoglobulin light chain expression, and all cells had the following characteristic surface phenotype: CD19+/CD5+/CD23+/CD20‐low+/CD22‐low+/CD43+/CD103−/CD11c− (Fig. S1).

**Figure 2:**
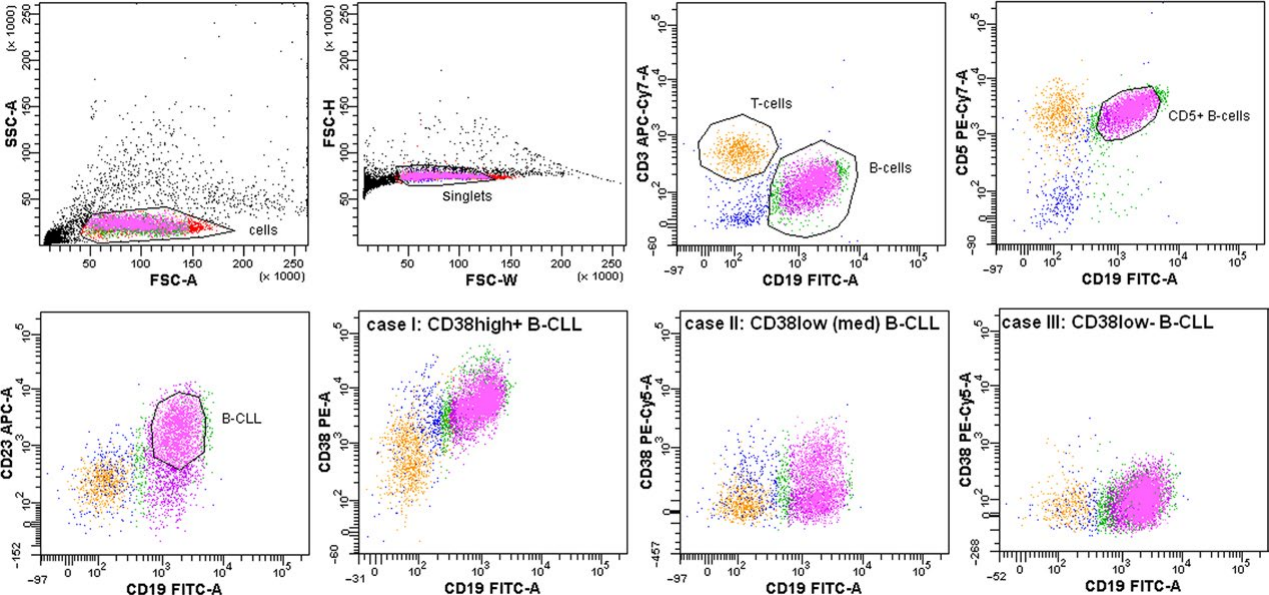
Flow cytometry and cell sorting strategy for B‐CLL cells. “Cells” are gated from debris according to forward and side light scatter characteristics; “singlets” are gated according to the FSC signal width/height ratio; “B‐cells” are gated based on CD19 fluorescence; B‐CLL cells are further gated based on the CD3‐CD19+CD5+CD23+ cell phenotype; the gating is sequential. The lower panel presents three different cases of B‐CLL with varying densities of surface CD38 (identified as high+, low [med], and low−).

The CD38 expression level in the B‐CLL lymphocytes varied substantially; however, the majority of the cases were CD38‐negative (the data regarding the population percentages and CD38 levels are summarized in Table S1). B‐CLL cells were sorted from PBMC samples (*n* = 20) according to the CD3−/CD5+/CD19+/CD23+ immunophenotype. An example of the sorting strategy of the B‐CLL population is presented in Figure 2.

**Figure 3:**
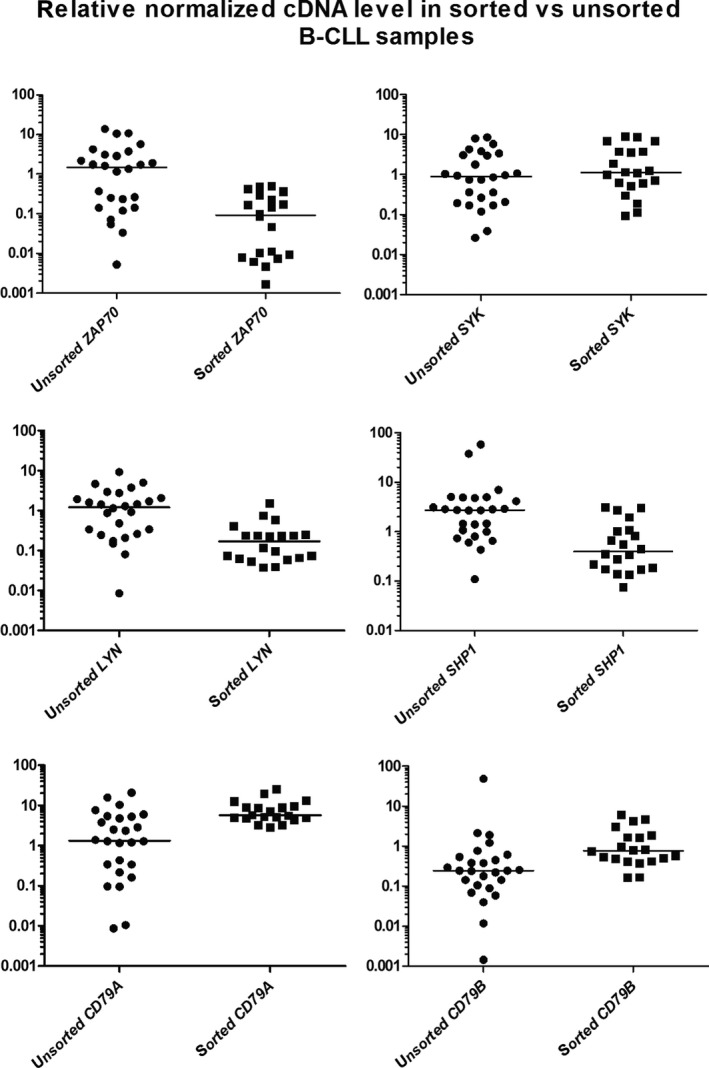
mRNA expression level of BCR signaling components in sorted and unsorted B‐CLL cells. Each measurement is performed in triplicate, and cDNA is normalized against the following three genes: *YWHAZ*,*HPRT1*, and *UBC*; bars represent the medians of each group.

In order to assess whether the sorting procedure changed the mRNA expression levels of the major signaling BCR‐associated proteins, 26 unsorted B‐CLL specimens were compared to 20 sorted B‐CLL specimens (Fig. 3). The differences in the mRNA expression levels between the sorted and unsorted samples were statistically significant for *ZAP70* (*P* < 0.0001), *LYN* (*P* < 0.0001), and *SHP1* (*P* < 0.001), but not for SYK. The most obvious difference was observed in *ZAP70* as its mRNA expression level decreased by two orders of magnitude in the sorted B‐CLL group (median 0.047 in sorted B‐CLL vs. 1.481 in unsorted B‐CLL, *P* < 0.001), which is very important considering its prognostic value. In the purified B‐CLL subpopulation, the mRNA expression levels of *SHP1* and *LYN* were decreased, and the expression levels of *CD79A* and *CD79B* were elevated (both 10‐fold) compared to the levels in the unsorted samples. Altogether, these observations highlight the problem of sample heterogeneity in mRNA expression measurements. While constitutive B‐cell mRNAs (such as *CD79A*,* CD79B*, and *SYK*) exhibit similar or decreased expression levels when normalized to housekeeping genes in the unsorted samples, the expression levels of other mRNAs (*ZAP70*,* SHP1*, and *LYN*) may be biased and, thus, “upregulated” due to the presence of T‐cells in the unsorted samples. Here, we demonstrate the statistical error that is introduced by sample heterogeneity.

Then, the mRNA expression levels of signal molecules in the sorted normal T‐cells, different populations of normal B‐cells, and malignant B‐CLL lymphocytes were assessed.

### Expression of signal molecules in normal T‐cells

**Figure 4:**
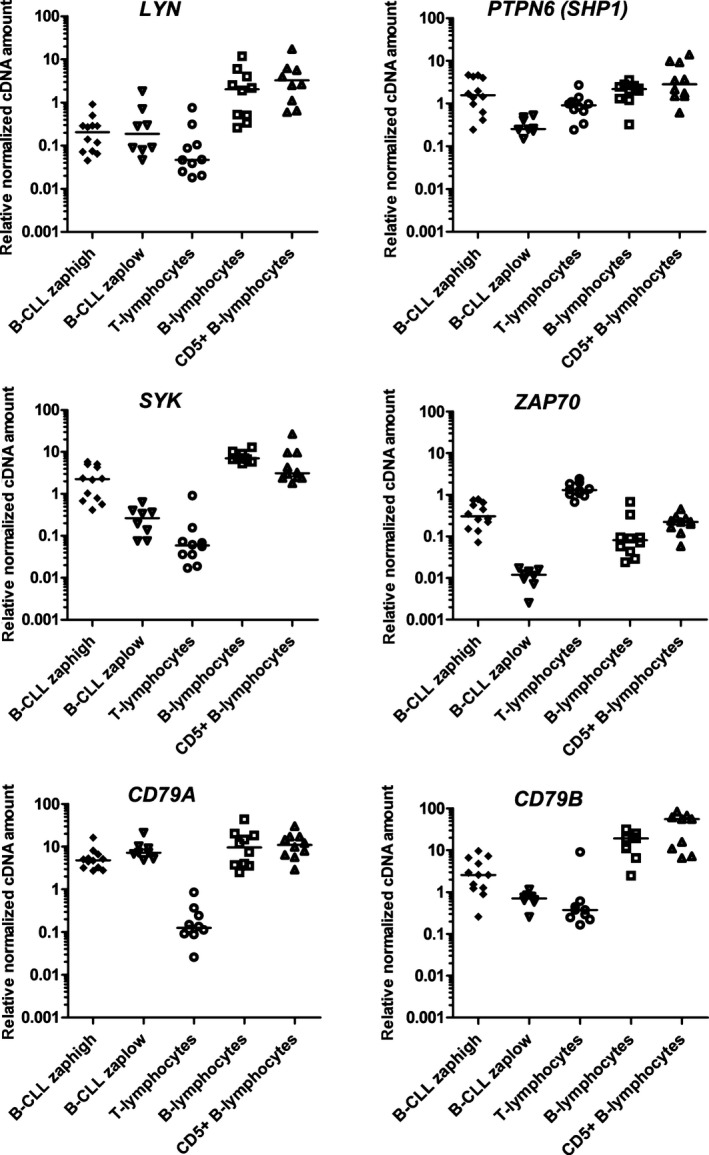
mRNA expression level of BCR components in normal and malignant lymphocytes. Each measurement is performed in triplicate, and cDNA is normalized against the following three genes: *YWHAZ*,*HPRT1*, and *UBC*.

T‐cells have the lowest mRNA expression levels of *LYN* (median 0.01), *SYK* (median 0.06), *CD79A* (median 0.13), and *CD79B* (median 0.37) (Table 1, Fig. 4). Because Zap‐70 tyrosine kinase is a normal counterpart of the T‐cell receptor, *ZAP70* was upregulated in the T‐cells compared to all other groups (median 1.3). Simultaneously, we observed lower levels of *SHP1* (*P* < 0.05) mRNA, even though it is a conventional tyrosine phosphatase that blocks downstream TCR signaling.

**Table 1:** mRNA expression levels of B‐cell receptor components in normal and malignant lymphocytes

	Lyn	SHP1	Syk	ZAP70	CD79a	CD79b
B‐CLL	0.05–1.84	0.01–4.63	0.08–5.79	0.002–0.78	0.25–21.40	0.19–12.97
Median	0.24	0.53	0.68	0.07	5.22	1.15
B‐cells	0.35–3.49	0.33–3.56	5.32–12.97	0.02–0.68	2.50–43.76	2.47–31.57
Median	0.68	2.17	7.03	0.08	9.63	19.18
CD5+ B‐cells	0.60–17.28	0.61–14.00	1.80–26.81	0.06–0.45	2.90–30.00	6.50–84.25
Median	2.62	2.81	3.12	0.22	11.04	55.72
T‐cells	0–0.09	0.24–2.70	0.02–0.91	0.66–2.42	0.03–0.85	0.17–9.10
Median	0.01	0.91	0.06	1.3	0.13	0.37

### Expression of B‐cell receptor components in CD5‐high and CD5‐low normal B‐cells

One of the aims of our study was to describe the expression profiles of the BCR signaling components in nonmalignant CD5‐high B‐cells and compare these profiles to those of normal B‐cells from peripheral blood. The expression level of *SYK* mRNA in the CD5‐high B‐cells from human tonsils was similar to that in the normal CD5‐low B‐cells (Table 1), although the variation coefficient in this group was several fold higher (CV = 31.7% for CD5‐low B‐cells vs. 119.4% for CD5‐high B‐cells). The differences in the mRNA expression levels of all other genes, except for *ZAP70*, in the CD5‐high and CD5‐low B‐cells were statistically insignificant. The expression of *ZAP70* in the CD5‐high B‐cells was more than 10‐fold higher than that in the CD5‐low B‐lymphocytes (median 1.3 vs. 0.08, respectively, *P* = 0.04). Fifteen samples of human tonsils were immunofluorescently stained for intracellular Zap‐70, and eight samples contained Zap‐70‐positive B‐cells (data not shown).

### Expression of signal molecules in sorted B‐CLL lymphocytes

*CD79A* was expressed at nearly the same level (range 4.83–11.04) in all types of B‐cells, including the malignant B‐CLL lymphocytes. As expected, the mRNA levels of *CD79B* and *SYK* in the B‐CLL group was compared to that in the normal CD5‐high and CD5‐low B‐cells (*P* < 0.05, see Table 1). *SHP1* mRNA was decreased in the B‐CLL samples to the same extent as that in the T‐cells compared to that in the normal B‐cells. The range of the expression of *ZAP70* in the B‐CLL group varied dramatically – from 1 × 10^−5^ to 0.78 (median 0.07, Table 2). Interestingly, a clear division of the B‐CLL group into *ZAP70*‐high and ZAP70‐low subgroups (Fig. 4) was observed.

**Table 2:** mRNA expression levels of signaling molecules in the *ZAP70*‐low and *ZAP70*‐high subgroups in the B‐CLL dataset

	ZAP‐70	Lyn	Syk	SHP‐1	CD79a	CD79b
B‐CLL ZAP‐70 “high”	0.07–0.78	0.05–0.90	0.42–5.79	0.24–4.63	2.74–16.30	0.26–9.56
Median	0.31	0.20	2.25	1.57	4.83	2.57
B‐CLL ZAP‐70 “low”	0.002–0.02	0.05–1.84	0.08–0.63	0.15–0.53	4.87–21.40	0.25–1.15
Median	0.01	0.18	0.27	0.25	7.23	0.70

Thus, we separated the *ZAP70*‐low (below 0.02) and *ZAP70*‐high (range 0.07–0.78) subgroups and compared their levels of mRNAs (Table 2). In the B‐CLL *ZAP70*‐low subgroup, a statistically significant downregulation of *SYK*,* SHP1*, and *CD79B*, and an upregulation of *CD79A* mRNA levels (*P* < 0.05 for all datasets) were observed. Thus, the *ZAP70*‐high and ZAP70‐low subgroups of B‐CLL have different transcription profiles for all BCR signaling molecules, except for *LYN* tyrosine kinase gene, which tends to be downregulated in all B‐CLL lymphocytes compared to normal CD5‐high and CD5‐low B‐cells. The *ZAP70*‐low subgroup appears to have a unique expression profile because it does not resemble either the CD5‐high B‐lymphocytes from the tonsils or the CD5‐low lymphocytes from the peripheral blood (the differences were statistically significant for all mRNAs, except for *CD79A*).

### Differences in protein expression of signal tyrosine kinases in sorted B‐CLL cells

**Figure 5:**
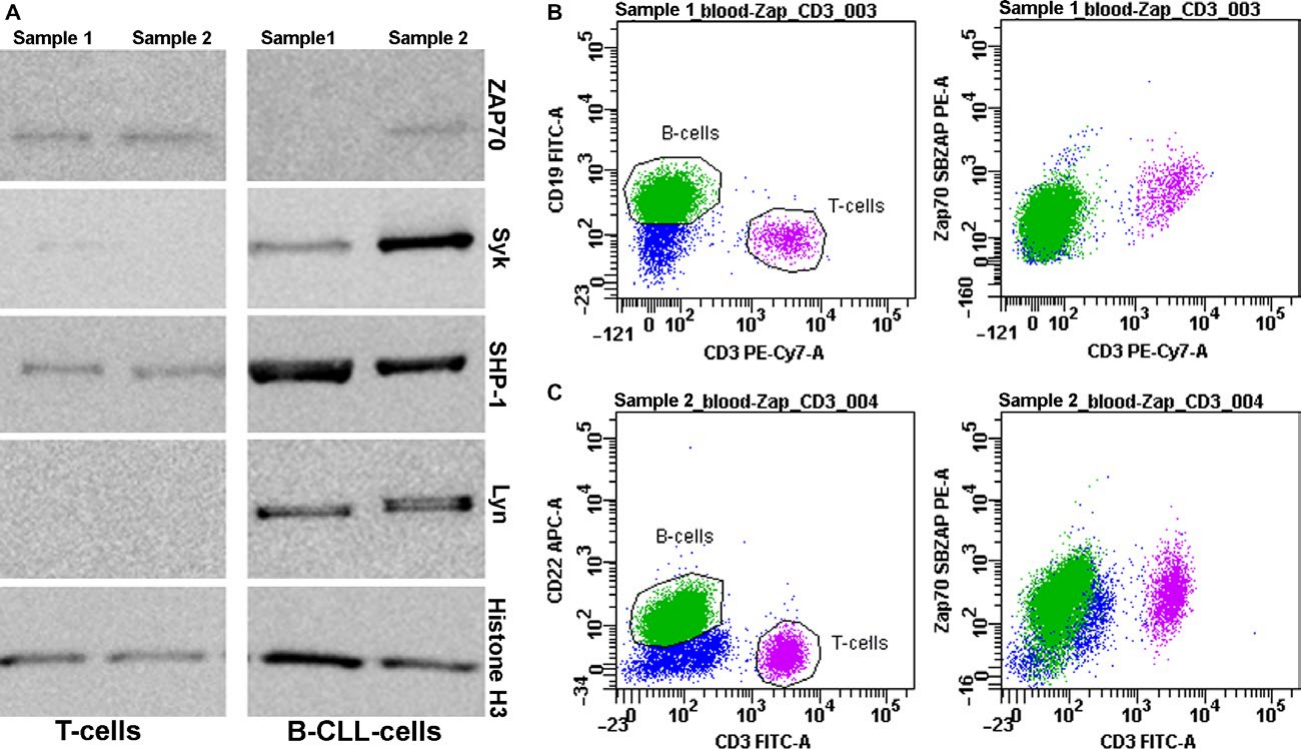
Protein expression level of BCR components in B‐CLL ZAP70‐high and ZAP70‐low representative cases. (A) Immunoblotting of signaling tyrosine kinases in ZAP70‐low sample (sample 1) and ZAP70‐high sample (sample 2), histone H3 was taken as a technical control. (B) Sample 1 (see A) analyzed by flow cytometry. Median fluorescence intensities of Zap70 in B‐CLL and T‐cell populations differ ×2.85‐fold; MFI(Zap70)T‐cells/MFI(Zap70)B‐CLL = 2.85. (C) Sample 2 (see A) analyzed by flow cytometry. Median fluorescence intensities of Zap70 in B‐CLL and T‐cell populations do not differ. MFI(Zap70)T‐cells/MFI(Zap70)B‐CLL = 1.

Finally, we have tested whether differences in mRNA expression can be confirmed on protein level and analyzed protein expression of BCR tyrosine kinases in *ZAP70‐*high and *ZAP70*‐low B‐CLL subsets compared to T‐cells as a control. In *ZAP70*‐low group, we confirmed a significant downregulation of Zap‐70 protein both by flow cytometry and immunoblotting. We also confirmed the downregulation of Syk tyrosine kinase for *ZAP70*‐low group on a protein level, and no differences in Lyn expression, the only discordance in transcriptional and translational levels was for SHP‐1 phosphatase that was uniformly expressed in all tested groups (Fig. 5).

## Discussion

FACS purification of cell populations for further qPCR analysis provides a powerful combination of methods that allows for gene expression profiling of both cancer cells and cancer‐associated cells. In this study, FACS sorting was performed to evaluate the quality of the B‐CLL gene expression data. We revealed statistically significant differences in the mRNAs of the BCR components in the sorted and unsorted B‐CLL samples, except for *SYK* tyrosine kinase. Thus, comparisons of unsorted B‐CLL samples and sorted normal B‐cells can be incorrect in signal transduction pathways’ studies because the admixture of T‐cells can alter the quantity of the tyrosine kinase cDNA. Using the FACS sorting method, we isolated the nonmalignant CD5‐high B‐cells and, for the first time, described the transcriptional profile of the BCR components.

CD5‐high normal B‐cells were first described in the fetal spleen, where they comprised 40–60% of the splenic B‐cells, but were infrequent in the adult spleen and fetal liver 19. In adult tonsils, CD5‐high B‐cells bear CD23 (low), IgM, and IgD 20. These B‐cells seed in the mantle zone of the lymphoid follicles, respond to polyclonal activation, have unmutated V‐kappa genes, and are not capable of producing natural polyspecific antibodies 21, 22, 23. It was shown that Zap‐70 was present in normal CD5‐high B‐cells from human tonsils but not in B‐cells from peripheral blood 24. Here, we show that CD5‐high and CD5‐low B‐cells have similar mRNA expression levels of *SYK*,* LYN*,* CD79A*,* CD79B*, and *SHP1* and confirm that *ZAP70* is the only upstream tyrosine kinase gene that allows for the discrimination among these cells based on the mRNA level.

The Zap‐70 protein is a 70‐kDa member of the Syk family of protein tyrosine kinases and was first identified as a crucial element for the downstream signaling from the T‐cell receptor 25. Similarly to Syk tyrosine kinase after B‐cell receptor (BCR) engagement, Zap‐70 is recruited to the phosphorylated immunoreceptor tyrosine activation motifs of the CD3 chains that are present in the T‐cell receptor, where it subsequently becomes phosphorylated and initiates several signaling cascades 26. The expression of the Zap‐70 protein and its correlation with the adverse prognosis in certain subsets of B‐lymphocyte‐derived malignancies prompted the analysis of its functions in a B‐lymphocyte setting. In B‐lymphocytes, Zap‐70 binds to the parts of the heterodimer CD79a/CD79b 27 and delays BCR internalization after IgM stimulation 28. In 2003, Weistner et al. found that Zap‐70 expression was a valuable prognostic marker in B‐CLL and that its expression is mainly correlated with known prognostic factors, such as the IGHV mutation status and percentage of CD38‐positive B‐cells 29. The low levels of Zap‐70 corresponded to indolent B‐CLL, and high Zap‐70 levels corresponded to a more aggressive disease or disease progression. Further studies confirmed the predictive value of this marker 30, 31, 32; however, the flow cytometry measurement of the Zap‐70 status is often inaccurate because of small differences between positive and negative populations 33 that can also be affected by the choice of antibody clone and gating procedures 34. Consequently, mRNA quantification for the determination of *ZAP70* expression was proposed 35. There were several attempts to measure *ZAP70* mRNA expression in normal and malignant tissues 36, 37, 38, 39. In those studies, the aim was to determine the correlation between *ZAP70* expression and other predictors of a pure outcome, such as the IGHV mutation status and CD38 expression. To normalize the *ZAP70* mRNA expression levels in tumor lymphocytes, *GUS*,* GAPDH*, and *PP1A* were proposed; however, each of these genes was used as a sole reference gene. To date, there are no studies including several housekeeping genes simultaneously for the accurate normalization of the *ZAP70* mRNA level. The best single‐gene SD‐based approach assumes that the gene expression levels are normally distributed throughout the sample set 40; however, the normality of the distribution in a dataset is rarely addressed in clinical studies. Accurate normalization with several reference genes increases the method's resolution and allows for resolving groups with only a 10‐fold difference in gene expression level as demonstrated in our previous work 18, 41. In current study, we used three housekeeping genes, namely, *YWHAZ*,* UBC*, and *HPRT1*, that have been shown to be optimal for PCR data normalization in different types of lymphoid tissue 18, Regarding *ZAP70* expression, the B‐CLL samples in our dataset can be divided into the following two groups (Table 2): low *ZAP70* mRNA expression (8 samples, normalized cDNA quantity lower than 0.04, median 0.01) and high *ZAP70* mRNA expression (12 samples, normalized cDNA quantity higher than 0.15, median 0.31). The *ZAP70* mRNA level in the first group corresponds to that in the CD5‐low normal B‐cells, and the *ZAP70* mRNA level in the second group corresponds to that in the CD5‐high B‐cells from the tonsils.

Overall, in the *ZAP70*‐low group, we observed a downregulated mRNA expression of *SYK*,* CD79B*, and *SHP1* compared to that in the *ZAP70*‐high group. Moreover, the *ZAP70‐*low B‐CLL cells demonstrated a lower mRNA expression of *SHP1*,* CD79B*,* SYK*, and *LYN* compared to the normal CD5‐high and CD5‐low B‐cells (*P* < 0.05 for all groups), thus demonstrating a unique mRNA profile that differs from any other type of B‐cells. This is the first report of the downregulation of a signal tyrosine kinase at the mRNA level. In 2009, Buchner et al. 14 showed an elevated mRNA expression level of *SYK* tyrosine kinase in B‐CLL cells, but these authors used the β‐glucuronidase gene as the sole reference gene in the ΔΔ*C*_t_ method, making their results questionable since nothing is known about the expression stability of this gene in normal and malignant lymphoid tissue.

Most of the recent BCR studies in B‐CLL cells focused on measuring the protein expression and phosphorylation level of these molecules, namely, Lyn and Syk kinases, which were reported to be constitutively active and upregulated at the protein level 12, 42. While Contri et al. 12 showed that Lyn is overexpressed at protein level, they reported no differences in mRNA expression of *LYN* measured by RT qPCR (data were not shown). Gobessi et al. 13 reported the substantial quantities of Y352‐phosphorylated *SYK* in most of B‐CLL samples, but there were no data on the overall quantity of *SYK* protein in B‐CLL cells compared to normal B‐lymphocytes. To date, there are no studies that directly compare mRNA and protein expression of BCR tyrosine kinases in B‐CLL cells and all the clinical studies that investigate the impact of BCR signaling pathway alterations in overall survival and clinical prognosis measure mRNA expression without confirmation of alterations at protein level 43. The possible reason is the difference in the detection limit of real‐time PCR as a semiquantitave method compared to qualitative measurement of protein expression by Western blotting.

The only exception is Zap‐70 molecule, that is routinely measured both on protein and mRNA levels with flow cytometry and real‐time PCR, respectively. For instance, assessing the samples from E2997 multicenter phase III trial, Kaplan et al. 44 showed that B‐CLL samples can be clearly distinguished into two subpopulations based on *ZAP70* expression. They have also showed that *ZAP70*‐high group samples demonstrate higher levels of antiapoptotic proteins (Bcl‐2, Puma, survivin), which is in line with our hypothesis on the defective apoptosis as a main result of BCR hyperactivation. Using high‐resolution immunophenotyping technology, this group also reported that increased expression of ZAP70 is correlated with low phosphorylation levels of Syk and Zap‐70 and that more aggressive clinical course of B‐CLL is related to higher levels of Zap‐70 more than BCR activation pathway 45, thus justifying the importance of functional measurements of tyrosine kinases both in fundamental and clinical research.

We have observed two distinct groups of B‐CLLs based on differential *ZAP70* mRNA expression. However, none of these groups fully correspond to any of the observed normal B‐cell populations in terms of investigated mRNA expression profiles. For instance, *ZAP70*‐low B‐CLLs have similarly decreased mRNAs of *SYK* and other BCR‐associated molecules. Such generally “low‐expressing” profile is highly uncommon for normal B lymphocytes and may correspond to “signaling‐deficient” B‐CLL cases, cited in literature previously 46. At the same time, *ZAP70*‐high B‐CLLs do not exhibit such obvious abnormalities but differ from normal B‐cells in expression of *CD79B* (which is common for all CLL samples) together with differences in *SYK* and *LYN* mRNA, which expression is similar to CD5‐high B‐cells and slightly downregulated compared to normal CD5‐low B‐lymphocytes. In our research, we have also confirmed the downregulation of Syk tyrosine kinase in *ZAP70*‐low group, and for the first time describe the correlation between the mRNA and protein expression of signaling tyrosine kinases in different subsets of B‐CLL samples, however, a quantitative measurement of phosphorylation pattern of signaling tyrosine kinases as a hallmark of their functionality is an important issue for further investigation.

**Figure 6:**
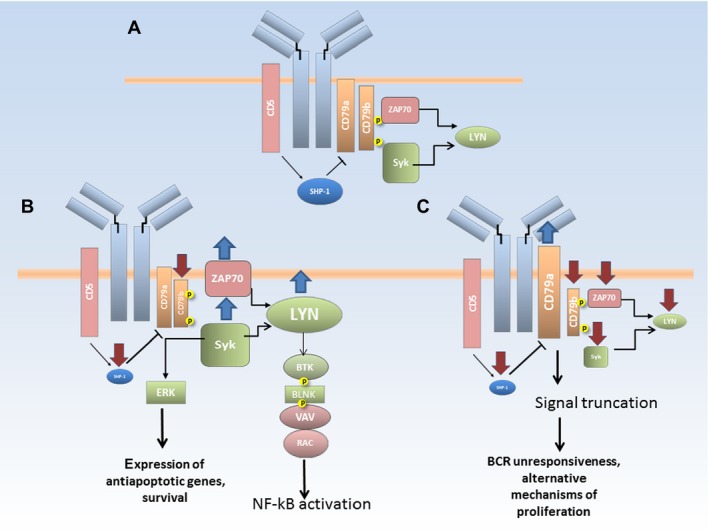
A model of BCR signaling pathways in normal B‐cells and CLL lymphocytes. (A) Structure of B‐cell receptor in normal B‐cells, characteristic features of BCR in CD5‐positive B‐cells are highlighted in red; (B) upregulation of key tyrosine kinases in B‐CLL cells with high level of ZAP70 expression leads to activation of major survival pathways; (C) downregulation of tyrosine kinases in CLL cells with low level of ZAP70 expression leads to signal truncation, which implies the existence of alternative signaling pathways that eventually promote proliferation.

Thus, in the *ZAP70*‐low group, we observed BCR signaling incompetence, which suggests that other mechanisms may override the inhibition of the B‐cell receptor pathway and eventually promote B‐cell proliferation. The evidence supporting this hypothesis is that a subset of B‐CLL cells with unresponsiveness to sIg ligation display constitutively activated extracellular signal‐regulated kinase ERK1/2 in the absence of Akt activation and the presence of increased NFAT transactivation 47. In contrast, the *ZAP70*‐high group displays an overexpression of all kinases involved in BCR signalosome formation together with the downregulation of *SHP1* inhibiting phosphatase gene (Fig. 6), which leads to the activation of the NFkB and AKT survival pathways and the expression of antiapoptotic proteins, such as mcl‐1 and bcl‐2, which have been shown to act as crucial players in defective apoptosis in B‐CLL cells 48, 49. Altogether, these data are relevant for differential therapeutic strategies and suggest a new approach of selecting therapeutic kinase inhibitors for the two B‐CLL subsets that could be easily distinguished by the *ZAP70* mRNA expression.

In conclusion, our observations suggest that it is necessary to perform a cell sorting prior to an analysis of gene expression to provide new insight into the regulation of the transcriptional levels of PTKs of B‐cell receptor in different B‐CLL subsets with high and low *ZAP70* expression. Our data are consistent with a model in which Zap‐70 is a key regulatory molecule, and its expression allows for the discrimination among different types of normal and malignant lymphocytes.

## Conflict of Interest

The authors report no financial or other conflict of interest relevant to the subject of this article.

## Supporting information


**Figure S1.** Immunophenotype of B‐CLL cells. The upper and middle panels show extracellular staining, the lower panel shows intracellular staining. Lymphocytes are gated according to forward and side light scatter characteristics; the malignant population defined by CD19+CD5+ cell phenotype is highlighted in blue. Kappa clone is confirmed on both extra‐ and intracellular staining.
**Table S1.** B‐CLL activation status based on surface CD38 expression.
**Table S2.** Primer sequences used in the study.
