# Supplementary material for: Comparison of the mRNA expression profile of B‐cell receptor components in normal CD5‐high B‐lymphocytes and chronic lymphocytic leukemia: a key role of ZAP70
Source: Cancer Med. 2017 Nov 10;6(12):2984–97. doi: 10.1002/cam4.1257 (PMC5727315; doi:10.1002/cam4.1257)
Supplement: Supplementary file 1 — Figure S1. Immunophenotype of B‐CLL cells. The upper and middle panels show extracellular staining, the lower panel shows intracellular staining. Lymphocytes are gated according to forward and side light scatter characteristics; the malignant population defined by CD19+CD5+ cell phenotype is highlighted in blue. Kappa clone is confirmed on both extra‐ and intracellular staining. Table S1. B‐CLL activation status based on surface CD38 expression. Table S2. Primer sequences used in the study. [file CAM4-6-2984-s001.pdf]

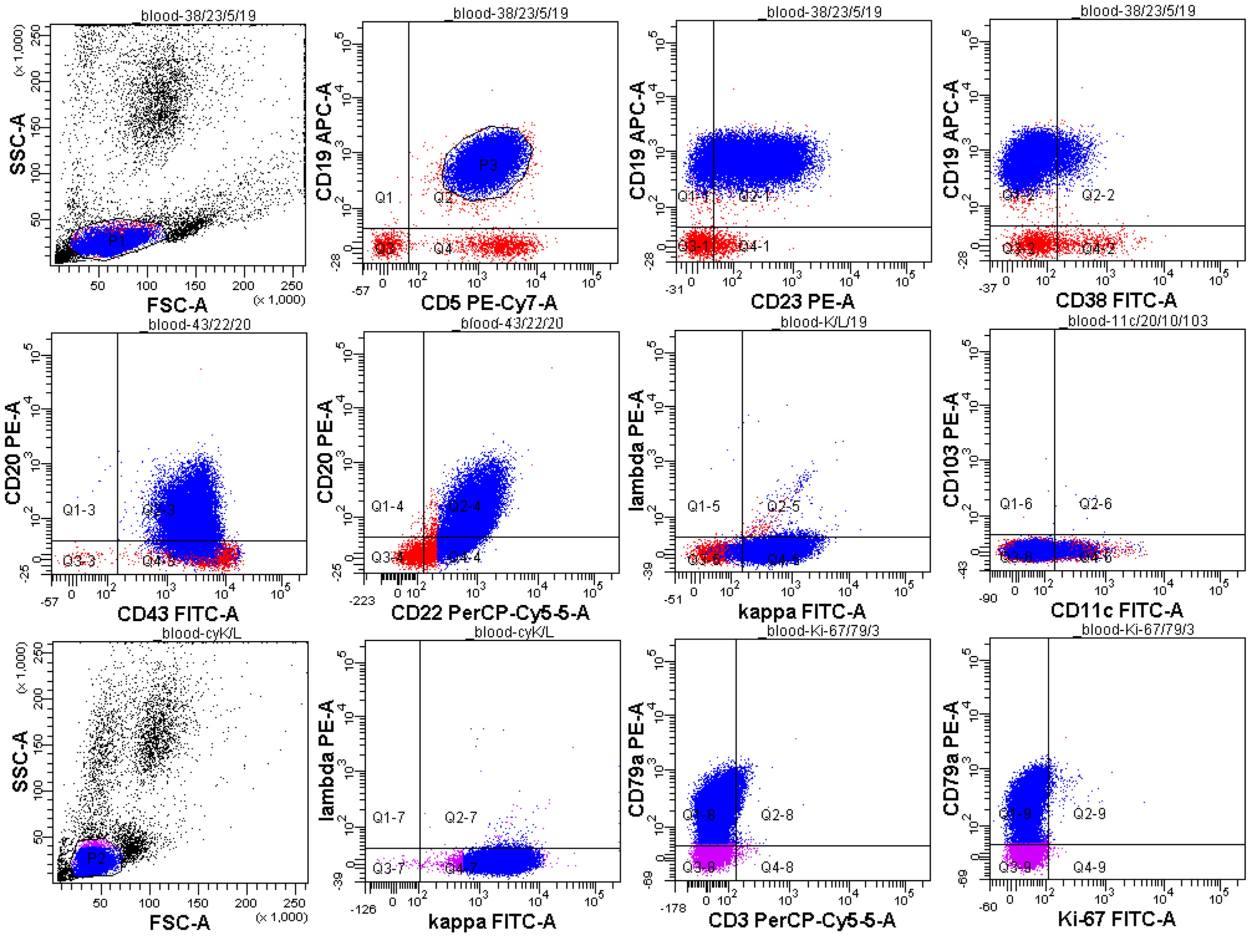

Figure S1. Immunophenotype of B-CLL cells. The upper and middle panels show extracellular staining, the lower panel shows intracellular staining. Lymphocytes are gated according to forward and side light scatter characteristics; the malignant population defined by CD19+CD5+ cell phenotype is highlighted in blue. Kappa clone is confirmed on both extra- and intracellular staining.

Table S1. B-CLL activation status based on surface CD38 expression.

Unsorted B-CLL			Sorted B-CLL (>98% purity)	
**№**	**Activation status**	**% of lymphocytes**	**№**	**Activation status**
1	CD38 low	64	1	CD38 low
2	CD38 low	14	2	CD38 low
3	CD38 low	63	3	CD38 low
4	CD38 low	71	4	CD38 low

5	CD38 low	94	5	CD38 low
6	CD38 low	45	6	CD38 low
7	CD38 low	62	7	CD38 low
8	CD38 low	53	8	CD38 low
9	CD38 low	84	9	CD38 low
10	CD38 low	33	10	CD38 low
11	CD38 low	56	11	CD38 low
12	CD38 low	68	12	CD38 low
13	CD38 low	44	13	CD38 low
14	CD38 low	38	14	CD38 low
15	CD38 low	32	15	CD38 low (med)
16	CD38 low	90	16	CD38 low (med)
17	CD38 low	56	17	CD38 low (med)
18	CD38 low	50	18	CD38 low (med)
19	CD38 low	65	19	CD38 high+
20	CD38 low (med)	64	20	CD38 high+
21	CD38 high+	85		
22	CD38 high+	30		
23	CD38 high+	95		
24	CD38 high+	92		
25	CD38 high+	96		
26	CD38 high+	63		

Table S2. Primer sequences used in the study

Gene	sense	antisense
UBC	5’-ATT TGG GTC GCG GTT CTT G-3’	5’-TGC CTT GAC ATT CTC GAT GGT-3
HPRT1	5’-TGA CAC TGG CAA AAC AAT GCA-3’	5’- GGT CCT TTT CAC CAG CAA GCT-3’
YWHAZ	5’- ACT TTT GGT ACA TTG TGG CTT CAA-3’	5’- CCG CCA GGA CAA ACC AGT AT-3’
LYN	5’- TCC TGA AGA GCG ATG AAG GT-3’	5’- CTG CCT TTT CTT TCC AGC AC - 3’

SYK	5’- TTC GGA CTC TCC AAA GCA CT - 3’	5’- TCA TCC CTC GAT ATG GCT TC - 3’
ZAP70	5’- CCA GAG GAG CTC AAG GAC A - 3’	5’- CCA CGT CGA TCT GCT TCT T - 3’
SHP1	5’ – CCT GGA GAC TTC GTG CTT TC – 3’	5’ – TCG GAC TCC TGC TTC TTG TT - 3’
CD79A	5’ – GGG CAA CGA GTC ATA CCA – 3’	5’ – AGC TTC TCG TTC TGC CAT – 3’
CD79B	5’ – GTC ATG GGA TTC AGC ACC TT – 3	5’- GCA GCG TCA CTA TGT CCT C - 3’
